# Tetracycline-grafted mPEG-PLGA micelles for bone-targeting and osteoporotic improvement

**DOI:** 10.3389/fphar.2022.993095

**Published:** 2022-09-14

**Authors:** Yunduan Que, Yuhang Yang, Hajra Zafar, Dongming Wang

**Affiliations:** ^1^ Department of Orthopedics, Nanjing Gaochun People’s Hospital, Gaochun Economic Development Zone, Nanjing, China; ^2^ School of Pharmacy, Jiangsu University, Zhenjiang, China; ^3^ School of Pharmacy, Shanghai Jiao Tong University, Shanghai, China

**Keywords:** MPEG-PLGA, tetracycline, astragaloside IV, osteoporosis, bone targeting

## Abstract

**Aim:** We aimed to create a nano drug delivery system with tetracycline (TC)-grafted methoxy poly-(ethylene-glycol)‒poly-(D, L-lactic-co-glycolic acid) (mPEG‒PLGA) micelles (TC‒mPEG‒PLGA) with TC and mPEG‒PLGA for potential bone targeting. Prospectively, TC‒mPEG‒PLGA aims to deliver bioactive compounds, such as astragaloside IV (AS), for osteoporotic therapy.

**Methods:** Preparation and evaluation of TC‒mPEG‒PLGA were accomplished via nano-properties, cytotoxicity, uptake by MC3T3-E1 cells, ability of hydroxyapatite targeting and potential bone targeting *in vivo,* as well as pharmacodynamics in a rat model.

**Results:** The measured particle size of AS-loaded TC‒mPEG‒PLGA micelles was an average of 52.16 ± 2.44 nm, which exhibited a sustained release effect compared to that by free AS. The TC‒mPEG‒PLGA demonstrated low cytotoxicity and was easily taken by MC3T3-E1 cells. Through assaying of bone targeting *in vitro* and *in vivo*, we observed that TC‒mPEG‒PLGA could effectively increase AS accumulation in bone. A pharmacodynamics study in mice suggested potentially increased bone mineral density by AS-loaded TC‒mPEG‒PLGA in ovariectomized rats compared to that by free AS.

**Conclusion:** The nano drug delivery system (TC‒mPEG‒PLGA) could target bone *in vitro* and *in vivo*, wherein it may be used as a novel delivery method for the enhancement of therapeutic effects of drugs with osteoporotic activity.

## 1 Introduction

Osteoporosis is considered a metabolic and systemic disease of bone with characteristics, such as decreased mass, increased fragility, and micro-structural disruption of bone, which culminates in increased fractures (Viprey et al., 2020). Usually, it is classified into primary osteoporosis (which includes senile, postmenopausal, and idiopathic osteoporosis) and secondary osteoporosis, which is mainly caused by disease and drug abuse (e.g., glucocorticoids) (Kim et al., 2016; Luo et al., 2019). Early clinical symptoms and signs of osteoporosis have been identified. Pathophysiologically, fracture coupled with complications caused by osteoporosis can lead to serious consequences, such as chronic lower back pain, impaired functioning, depression, and mobility disorders (Liu and Xu, 2018). Fractures relating to osteoporosis occur annually in roughly 1.5 million individuals worldwide, amid the incidence of osteoporosis being ranked sixth in the world’s common and frequently occurring diseases (Amin et al., 2020). As the aged population increases globally, osteoporosis-caused fractures are regarded as the major public health problem worldwide. Therefore, effective options for osteoporotic prevention and treatment are particularly needed.

Currently, bone formation promoters (e.g., fluoride, strontium salt, and parathyroid hormone) and bone resorption inhibitors (e.g., estrogen, calcitonin, and bisphosphonates) are used in the clinical treatment of osteoporosis (De Martinis et al., 2020; Estell and Rosen, 2021). Promotion of bone formation reduces bone loss by inhibiting osteoclasts, but it does not promote activation and differentiation of osteoblasts. They can only slow down the symptoms of osteoporosis without reversing bone mass or repairing damaged bone tissue (Quach et al., 2018; Chellaiah et al., 2020). Besides, pharmacotherapy has many problems, including a long cycle of therapy, poor compliance by patients, high cost, and adverse drug reactions (Li et al., 2020).

Tetracyclines (TCs) are usually used as a class of broad-spectrum antibiotics in clinical settings, but they have been found to exhibit good application prospects in the treatment of osteoporosis (Xie et al., 2017; Xie et al., 2018; Wang et al., 2020). Thus, TC demonstrates excellent targeting ability for bone tissue by complexing with calcium ions in hydroxyapatite, which is the main component of bone, albeit having little effect on non-bone tissues (Wang et al., 2015). Therefore, TC may be used as a potential compound for bone targeting. Usually, hydroxyapatites are exposed to blood during bone diseases, thus we can explore development of bone targeting nanoparticles, wherein tetracycline can serve as a ligand on the surfaces such nanoparticles. Through this means, tetracycline can be delivered specifically to bone tissues via its adsorption effect on hydroxyapatite, the mineral constituent of bone tissue (Low and Kopeček, 2012).

Astragaloside IV (AS), as one of the main active components of *Astragalus membranaceus*, is a natural antioxidant with anti-inflammatory, anti-aging, and immune regulatory activities (Jiang et al., 2019; Xu et al., 2021). Existing research has found that AS could suppress osteoclastogenesis induced by RANKL via inhibition of the ERK pathway (Li et al., 2015). Additionally, Ou et al. observed a substantial association between AS and various signaling pathways of MAPK, Fox-O, and PI3K-Akt, which are involved in osteoporosis. An earlier report has posited that AS is stably bound to *AKT1, PIK3CA,* and *SRC*, which may be considered as the hub genes for osteoporotic treatment (Ou et al., 2021).

This novel drug delivery approach has many advantages, including targeting to specific tissue, increased biocompatibility, and decreased side effects (Izham et al., 2019; Li et al., 2021; Sun et al., 2021). These advantages have increased the attention of researchers to nano drug delivery systems in the pharmaceutical field in recent years. As a biodegradable biopolymer, poly (D, L-lactic-co-glycolic acid) (PLGA) has shown great biocompatibility. PLGA has been approved by the Food and Drug Administration as one of the commonly used pharmaceutical excipients, wherein it is widely used for the preparation of microspheres, microcapsules, nanoparticles, and implanted scaffolds (Zhong et al., 2010; Zhu et al., 2012; Kefayat and Vaezifar, 2019). Besides, the PLGA nano drug delivery system has been applied in drug-controlled release, targeting organs and delivery of protein, as well as peptide and gene oral delivery (Liu et al., 2010; Kasiewicz and Whitehead, 2017). In particular, for some low oral bioavailability drugs with poor stability in the gastrointestinal tract, the PLGA nano drug delivery system was considered an effective solution (des Rieux et al., 2006). Therefore, modification of PLGA for targeted drug delivery to bone may be a useful way for osteoporotic therapy.

In the present study, TC and PLGA were covalently linked to the two ends of polyethylene glycol (mPEG) with carboxyl groups via amide linkage before self-assembling to form micelles in water, namely TC‒mPEG‒PLGA and AS-loaded (TC‒mPEG‒PLGA/AS). The droplet size, polymer dispersity index (PDI), transmission electron microscope (TEM), efficiency of encapsulation (EE), and loading of drug (DL) into the micellar system were applied appropriately to characterize TC‒mPEG‒PLGA/AS. The dialysis bag method was utilized to investigate *in vitro* drug release behavior. Investigation of cytotoxicity and cellular uptake was accomplished with MC3T3-E1 cells. Hydroxyapatite was used as an *in vitro* bone analogue model to ascertain the bone affinity of TC‒mPEG‒PLGA/AS. Additionally, an *in vivo* bone targeting assay was performed via live fluorescent imaging. The ovariectomized female rats were used as the osteoporotic animal model to evaluate the anti-osteoporotic effect of TC‒mPEG‒PLGA/AS. We expected that TC‒mPEG‒PLGA could increase AS accumulation in bone, amid improvement of anti-osteoporotic activity.

## 2 Materials and methods

### 2.1 Ethical compliance

The animal study was reviewed and approved by the Ethics Committee of Jiangsu University.

### 2.2 Materials

Zhenzhun Biomaterial Co., Ltd. (Shanghai, China) supplied mPEG (molecular weight of 2000 Da)/PLGA 50/50 (molecular weight of 18,000 Da). PEG-bis-carboxymethyl (molecular weight of 500 Da) was bought from Kaizheng United Medical Technology Co., Ltd. (Beijing, China). Sigma-Aldrich Co., (St Louis, MO-USA) provided N, N′-dicyclohexyl-carbodiimide (DCC), TC, 3-(4,5-dimethylthiazol-2-yl)-2,5-diphenyl-tetrazolium bromide (MTT), 4-dimethyl-aminopyridine (DMAP), N, N-dimethylformamide (DMF), and fluorescein isothiocyanate (FITC). We obtained an assay kit for bicinchoninic acid (BCA) protein from Beyotime Inst. Of Biotech (Haimen, Jiangsu-China). Moreover, minimum essential medium alpha (MEM α) was bought from HyClone Company (Beijing, China), while Gibco BIL Company (Gibco, United States) supplied serum of fetal bovine (FBS). All the other reagents were commercially and analytically pure.

The Laboratory for Animal Center at Jiangsu University (Zhenjiang, China) provided animals, including US Institute of Cancer Research (ICR) mice (18 ± 2 g) and female Sprague‒Dawley (SD) rats (200 ± 20 g). Approval of the protocol for animal experiments was given by the University Ethical Committee for Experimental Animal Use, wherein it was adapted to guidelines for the Use and Care of Animals in the Laboratory.

### 2.3 Synthesis and characterization of TC‒mPEG‒PLGA

Under conditions of sonication, we dissolved TC (13 mg, 0.03 mM) in DMF (5 ml) before the addition of DMAP (1.2 mg, 0.01 mM), DCC (7 mg, 0.036 mM) and PEG-bis-carboxy-methyl (18 mg, 0.03 mM) in an ice bath under stirring for 30 min. Transfer (for 24 h) of the reaction mixture to ambient temperature was accomplished under a nitrogen atmosphere. Later, mPEG‒PLGA (500 mg, 0.025 mM) was added in an ice bath for 30 min before transfer under the same conditions stated above. The solvent of the reaction was dried via rotovator under reduced pressure. Dialysis of reaction products was accomplished with a dialysis membrane (molecular weight cutoff [MWCO] of 7.0 × 10^3^) in ultrapure water for 2 days. The final product, TC‒mPEG‒PLGA, was obtained after lyophilization.

The structure of the synthesized TC‒mPEG‒PLGA was confirmed by ^1^H nuclear magnetic resonance (^1^H-NMR) spectra. TC and TC‒mPEG‒PLGA were dissolved in 0.5 ml of deuterium dimethyl sulfoxide (DMSO) and maintained at a final concentration of 20 mg/ml for the assessment of its nuclear magnetic spectrum.

### 2.4 Critical micellar concentration estimation

Estimation of the critical micellar concentration (CMC) of mPEG‒PLGA and TC‒mPEG‒PLGA was carried out with a fluorescence photometer. The steps were as follows: 6.0 mg of pyrene was dissolved in 500 ml of acetone to obtain a 6 × 10^–5^ mol/L solution, before it was sealed away from light for later use. We accurately prepared a 0.2 mg/ml aqueous solution of polymer micelles and performed gradient dilution to form gradient concentrations (1 × 10^–1^, 5 × 10^–2^, 3 × 10^–2^, 2 × 10^–2^, 1 × 10^–2^, 5 × 10^–3^, 2 × 10^–3^, 1 × 10^–3^, and 1 × 10^–4^ mg/ml) before it was sealed as described above. Later, 40 μL (6 × 10^–5^ mol/L) of pyrene solution was placed into the centrifuge tube and blow dried with nitrogen, before dissolution of the pyrene solution with various concentrations of micelle solution, yielding a final pyrene concentration of 6 × 10^–7^ mol/L. The tubes were shaken for 4 h at 37°C. A fluorescence spectrophotometer was used to scan the excitation and emission spectra of pyrene. The ratio of the first strong peak (375 nm) to the third (385 nm) (I_1_/I_3_) was used as the vertical coordinate, with the logarithm of the polymer concentration (logC) as the horizontal coordinate. Later, CMC was estimated using a concentration that corresponded to an inflection point.

### 2.5 Preparation of mPEG‒PLGA/AS and TC‒mPEG‒PLGA/AS

We placed AS (10 mg) and mPEG‒PLGA or TC‒mPEG‒PLGA (80, 100, 120, or 140 mg) in the conical flask with 100 ml of methanol. Afterwards, methanol was evaporated by rotovator to obtain a pale milky white film. Next, we placed the flask in an ice bath before vacuum drying to completely remove the residual methanol. The thin film was hydrated for 30 min at 37°C by gently shaking it with 30 ml of distilled water. Then, 5 min of ultrasonic treatment at ambient temperature was performed before filtration with a microporous membrane (0.22 μm) to obtain mPEG‒PLGA/AS or TC‒mPEG‒PLGA/AS.

### 2.6 Characterization of TC‒mPEG‒PLGA/AS

#### 2.6.1 Morphology observation

Observation of morphological characteristics of mPEG‒PLGA, mPEG‒PLGA/AS, TC‒mPEG‒PLGA, and TC‒mPEG‒PLGA/AS was accomplished with TEM (H-7650, Hi-tachi Ltd., Tokyo, Japan). Before phosphotungstate (2%) staining of the aforementioned micelles, we placed them onto a copper grid (Yang et al., 2019).

#### 2.6.2 Determination of droplet size and PDI

Droplet size and PDI were measured via dynamic light scattering analysis using a Zetasizer Nano-ZS model (Malvern Instruments Nordic AB, Skallestad, Norway). The measurement was performed at a temperature of 25°C and a scattering angle of 90° (Wang et al., 2019a).

### 2.7 Analysis using high performance liquid chromatography

In-house *in vitro* analysis of AS was performed with a Shimadzu high performance liquid chromatography (HPLC) machine (LC-20-AD, Shimadzu-Japan) equipped with an ultraviolet detector and a Purospher^®^ RP18 column (5 μm, 4.6 × 250 mm, Merck KGaA-Germany). We analyzed AS at acetonitrile (65, v)/water (35, 5) mixture as mobile phase, 1.0 ml/min as flow rate, 30°C as temperature of the column, 205 nm as detection wavelength for 10 min, and 20 μL of injection volume of sample. For *in vivo* detection, the internal standard (IS) was a baicalein (20 μg/ml) solution, wherein ethyl acetate was employed to extract AS and IS from plasma (Zhen et al., 2020).

### 2.8 Estimation of encapsulation efficiency and drug loading

An ultrafiltration centrifuge tube (molecular weight of 2,500) was used to determine encapsulation efficiency (EE) and drug loading (DL). In brief, the micelles were precipitated in the tube under low temperature and high-speed centrifugation. We added 10 times the volume of methanol to disrupt the structure of precipitated micelles before HPLC was applied to detect drug concentration (C_encapsulated_). The concentration of drug that was not precipitated in the tube (C_unencapsulated_) was obtained via the same technique. The total weight of micelle (M) was achieved by freeze-drying for 48 h. The EE and DL were calculated using equation (Zhang et al., 2019):

$$EE=\frac{W_{\mathrm{encapsulated}}}{W_{\mathrm{encapsulated}}+W_{\mathrm{unencapsulated}}}\times 100\%$$ (1)

$$DL=\frac{W_{\mathrm{encapsulated}}}{M}\times 100\%$$ (2)

The W_encapsulated_ and W_unencapsulated_ represent the quantity of encapsulated AS and unencapsulated AS in micelles, respectively. M represents the quantity of total micellar excipients.

### 2.9 Investigation of AS release *in vitro*

The *in vitro* release behaviour of AS and TC‒mPEG‒PLGA/AS was investigated with the dialysis bag method. The AS suspension or TC‒mPEG‒PLGA/AS containing 50 μg of AS was placed in dialysis bags (molecular weight of 14 K; Spectrum Labs, Laguna Hills, CA, United States) in phosphate-buffered saline (PBS, 15 ml, pH 7.4) with 0.5% Tween 80. The experiment was conducted with an oscillating machine (ZRS-8G model, Tianjin Univ. Radio Factory, China) at a speed of 100 rpm and 37 °C. Withdrawal of dissolution medium for analysis was accordingly performed, while the same volume of fresh medium was added at preset time periods. Measurement of AS concentration for the calculation of the cumulative release ratio with HPLC was carried out. The release curve was drawn with the abscissa as time and the cumulative release ratio as the ordinate (Weng et al., 2019).

### 2.10 Cytotoxicity evaluation

We employed an MTT colorimetric assay to evaluate mPEG‒PLGA/AS and TC‒mPEG‒PLGA/AS cytotoxicity. At 5 × 10^4^ cells/mL density, plating of MC3T3-E1 cells in 96-well plates was performed before 24 h of culturing at 37°C and CO_2_ (5%). After complete adherent of cells, different concentrations of mPEG‒PLGA/AS and TC‒mPEG‒PLGA/AS (1, 5, 10, 25, 50, 100, 150, and 200 μg/ml) were added, while cells without AS treatment were set as a negative control. Following 48 h of culturing, we added an aliquot (20 μL) of MTT (5 mg/ml) to each well, followed by another 4 h of incubation. Later on, we removed the supernatant prior to the addition of DMSO (100 μL) to each well and 20 min of oscillation. Reading of absorbance (A) was accomplished at 570 nm with a micro-plate reader machine (Thermo Fisher-USA). Based on an earlier established equation (Stryjska et al., 2020), we calculated the cell viability.

$$I\%=\frac{A_{\mathrm{treat}}}{A_{\mathrm{control}}}\times 100\%$$ (3)

where I% represents cell viability. The A_treat_ and A_control_ represent the absorbance of the negative control and sample groups at 570 nm.

### 2.11 Investigation of uptake of micelles by MC3T3-E1 cells

Based on the previous studies (Wang et al., 2015), we designed the uptake of micelles by MC3T3-E1 cells. Plating of MC3T3-E1 cells in a 12-well plate was performed at the same density and condition as stated in Section 2.9. Afterwards, we subjected the cells to overnight incubation (37°C) with octadecylamine-FITC (ODA-FITC) loaded mPEG‒PLGA/AS and TC‒mPEG‒PLGA/AS (at 0.1, 1, and 5 μg/ml). After a preset time interval of 1, 3, or 24 h, we washed the cells with PBS before their collection. Later, we freeze-thawed the collected cells with dimethyl sulfoxide. Following a 10-min centrifugation at 10,000 rpm, the fluorescence spectrophotometer was used to measure the fluorescence of the supernatant, while the cellular protein content was determined using a bicinchoninic acid kit. The ratio of cellular uptake of fluorescent-loaded mPEG‒PLGA/AS and TC‒mPEG‒PLGA/AS was computed using the previous equation:

$$Uptake\;ratio\;\left(\%\right)=\frac{\;Ct}{{\mathrm{Ct}}_{0}}\times 100\%$$ (4)

where C_t0_ represents the initial concentration of ODA–FITC at time t_0_ and C_t_ denotes the intracellular concentration of ODA–FITC at time t.

### 2.12 Evaluation of hydroxyapatite-targeting ability

Bone targets (mPEG‒PLGA/AS, TC‒mPEG‒PLGA/AS, and free AS) containing AS (1 or 5 mg) were put into a clean test tube, and 80 mg of hydroxyapatite was added. The samples were shaken at 37°C for 0.5, 1, 2, and 4 h, before being centrifuged for 10 min at 8,000 rpm. The precipitates were dissolved with 10 ml of methanol prior to 5 min of sonication and 10 min of centrifugation at 12,000 rpm. Filtration of the supernatant was accomplished with a filtering membrane (0.22 μm). The concentration of AS in the supernatant was detected by HPLC. The adsorption ability of bone targets was calculated by the following equation (Yuan et al., 2020):

$$A\%=\frac{I_{a}}{I0}\times 100\%$$ (5)

where A% represents the adsorption ability of bone targets. The I_a_ denotes the amount of AS absorbed by hydroxyapatite. The I_0_ denotes the weight of AS in initial bone targets.

### 2.13 *In vivo* bone-targeting study

Live imaging was applied to study bone targeting ability *in vivo*. This was accomplished by labelling mPEG‒PLGA/AS and TC‒mPEG‒PLGA/AS (10 mg/kg) with lipophilic DiR dye before evaluation of bone targeting ability in ICR mice. Allocated mice in the two groups received mPEG‒PLGA/AS or TC‒mPEG‒PLGA/AS (10 mg/kg; *n* = 3) via the caudal vein. During the experiment, unrestricted access was given to the mice in terms of food and water. In predetermined time intervals (2, 4, and 8 h), the fluorescent signals in femurs were observed with the Maestro EX system of imaging *in vivo* (Research and Instrumentation of Cambridge, Inc., Woburn, MA-USA).

### 2.14 Estimation of pharmacokinetic parameters in the plasma

At random, we allocated the SD rats into free AS and TC‒mPEG‒PLGA/AS groups. Administration of free AS and TC‒mPEG‒PLGA/AS was carried out based on the weight (10 mg/kg) of the rats. Sampling of blood from the retro-orbital veins of rats at preset time intervals was performed after they had received the aforementioned dosage forms. To obtain serum, we stored the sampled blood for 30 min at 37°C prior to centrifugation at 3,700 rpm for 10 min. The following procedure has been depicted in Section 2.7. Based on observed values, we computed C_max_ as the maximum AS concentration in plasma and T_max_ as the time to attain C_max_. Analysis of other parameters, such as curves of area-under-concentration time (AUC) and mean-residence time (MRT), was accomplished with pharmacokinetic software (BAPP 2.3 version). Estimation of oral relative biological availability (RBA) was carried out as follows (Zhu et al., 2021):

$$RBA=\frac{{AUC}_{TC‒mPEG‒PLGA}}{{AUC}_{AS}}\times 100\%$$ (6)

The AUC_TC‒mPEG‒PLGA/AS_/AUC_AS_ denotes the AUC of TC‒mPEG‒PLGA/AS and free AS, respectively.

### 2.15 Tissue distribution

After 12 h of fasting, the ICR mice were allocated into two groups at random. The ICR mice received free AS and TC‒mPEG‒PLGA/AS (25 mg/kg dose of AS) via the tail vein. They were sacrificed at pre-designed periods of time (0.25, 0.5, 1, 2, and 4 h) before the collection of organs, viz.*,* femur, liver, heart, kidney, spleen, and lung. Afterwards, the organs were stored in physiological saline. Later, they were pulverized with high-speed shear to achieve a homogenate suspension. The concentration of AS in organ suspension was detected to observe the tissue distribution behavior of TC‒mPEG‒PLGA/AS (Zhang et al., 2017).

### 2.16 Osteoporotic studies

Female SD rats were adopted for the osteoporotic study. SD rats were randomly assigned to one of five groups (*n* = 5): sham operated control rats (SHAM), ovariectomized rats (OVX), estradiol receiving ovariectomized rats *via* intravenous route at 0.5 mg/kg/2 days dose (OVX + ES), AS receiving ovariectomized rats *via* intravenous route at 0.5 mg/kg/2 days dose (OVX + AS) and AS-TC‒mPEG‒PLGA receiving ovariectomized rats *via* intravenous route at 0.5 mg/kg/2 days dose (OVX + TC‒mPEG‒PLGA/AS) (Wang et al., 2015). All SD rats were anesthetized and bilaterally ovariectomized except those in the SHAM group, which underwent a sham operation. The ES, AS, and TC‒mPEG‒PLGA/AS were administered 30 days later amid continuation for 60 days. All SD rats were sacrificed, and their left femurs were collected. Detection of BMD was performed with a dual energy X-ray absorptiometric machine (Skyscan1174 X-Ray Microtomograph, Bruker, Belgium) (Zhang et al., 2020).

### 2.17 Histopathological examination

Right femurs were collected before being washed in pH 7.4 PBS and fixed in paraformaldehyde (4%) at 4°C for 24 h. We carried out embedment of fixed frozen samples in paraffin before sectioning (5 μm) the samples for staining with hematoxylin and eosin (H&E). Later, the slides were photographed under a microscope (Nikon, Japan) for histopathological examination (Wang et al., 2019b).

### 2.18 Statistical analysis

A mean and standard deviation were utilized to depict data that was derived from the experiments. In terms of statistics, the Student’s t test was employed to ascertain two group differences, wherein we accepted *p* < 0.05 as the level of significance.

## 3 Results and discussion

### 3.1 Synthesis and characterization of TC‒mPEG‒PLGA

**FIGURE 1:**
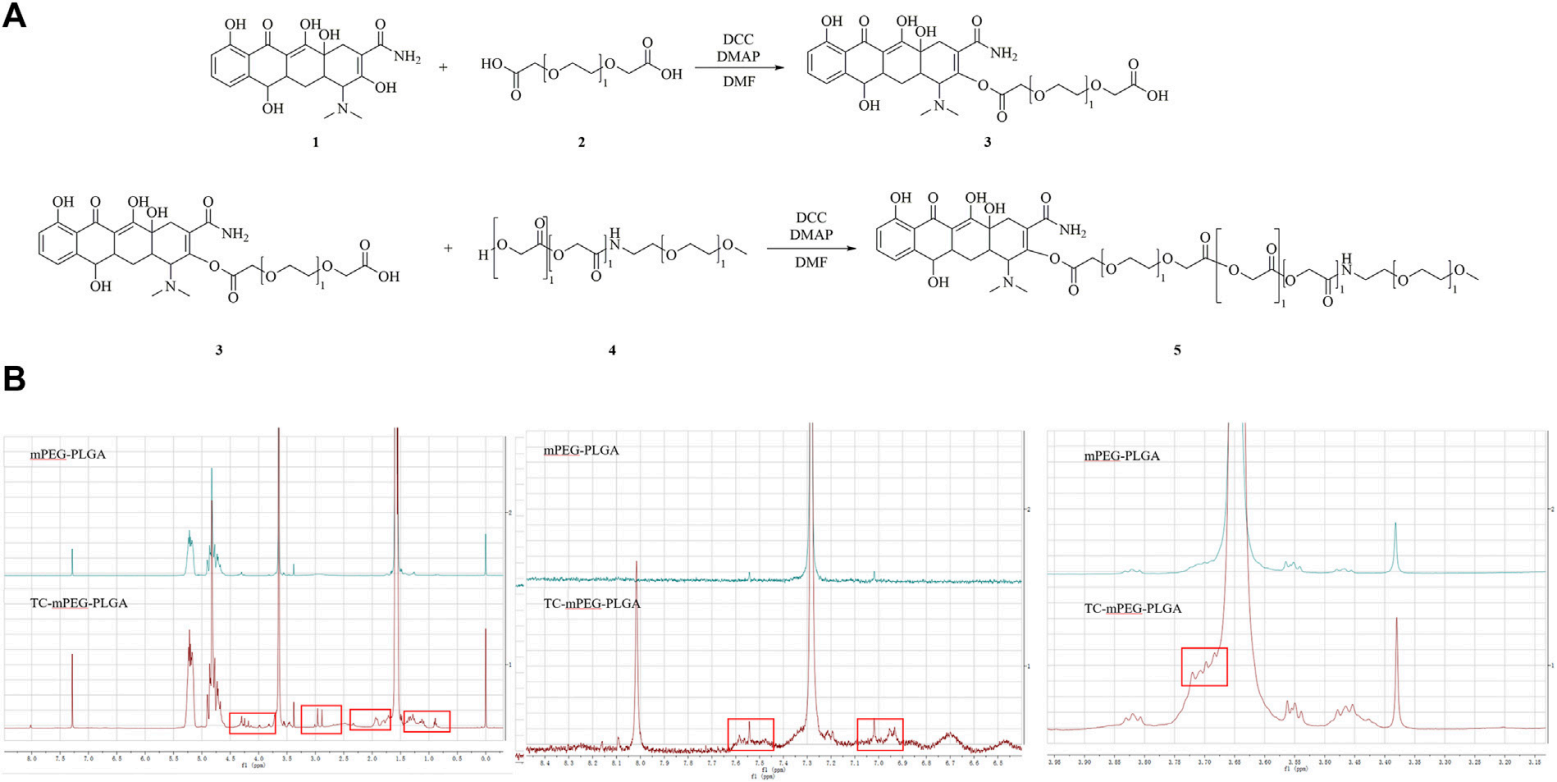
The chemical of TC-mPEG-PLGA and synthesis route of TC grafted mPEG‒PLGA micelles **(A)** and ^1^H-NMR spectra of TC and TC grafted mPEG‒PLGA micelles **(B)**. TC, tetracycline; DCC, N, N′-dicyclohexyl-carbodiimide; DMAP, 4-dimethyl-aminopyridine; DMF, N, N-dimethylformamide; mPEG‒PLGA, methoxy poly-(ethylene-glycol)‒poly-(D, L-lactic-co-glycolic acid).

The chemical structure of TC‒mPEG‒PLGA and the preparation process are illustrated in Figure 1A. ^1^H-NMR was used to confirm the structure of TC‒mPEG‒PLGA. By comparing the ^1^H-NMR spectrum of mPEG‒PLGA and TC‒mPEG‒PLGA, the structure of the graft TC‒mPEG‒PLGA was analyzed. As shown in Figure 1B, the ^1^H-NMR chemical shift that belongs to TC is marked in red and confirms the successful linkage of TC and mPEG‒PLGA. In this experiment, DMAP was used to catalyze the esterification and dehydration reactions of the free hydroxyl groups of mPEG and the carboxyl moiety of PLGA copolymer. The reaction was performed under anhydrous conditions to improve reactant activity with a dehydrating agent (DCC). The synthesis of the TC‒mPEG‒PLGA copolymer was accomplished by reacting the carboxyl moiety of mPEG in DDC with the amino group of TC. The TC‒mPEG‒PLGA copolymer was an amphiphilic compound, wherein mPEG acted as hydrophilic groups, while PLGA served as hydrophobic groups (Li and Zhao, 2019). The TC‒mPEG‒PLGA self-assembled into micelles in water on account of the hydrophobic segments segregating from water to form an inner core and the hydrophilic segments orientating outward to contact with water. The CMCs of mPEG-PLGA and TC-mPEG-PLGA polymers in water were 9.516 μg/ml and 9.331 μg/ml, respectively, which were relatively low. This suggest that the polymers can sell assemble to form stable micellar systems upon dilution in biological media (Lu et al., 2018). It is suggested that at concentration lower than the CMC, there is accumulation of polymers at the interface of air-water until saturation of both the bulk and interface. Subsequently, water molecule is removed through micellar formation into aqueous phase bulk, which increases the entropy of the system, therefore entropically favoring this process (Adams et al., 2003; Kedar et al., 2010). As a valuable index of stable micelles and ability of micellization, CMC at lower value ensure ease of micellar preparation with colloidal stability. The value of CMC is evidently proven by experimental data, wherein it varies with copolymer composition, amid an increase in hydrophobic block size result in exponential decrease in CMC values (Sun et al., 2016; Wakaskar, 2017). It is evident from the experimental data that the CMC value varies with the composition of the copolymer.

### 3.2 Determination of TC‒mPEG‒PLGA/AS characteristics

**FIGURE 2:**
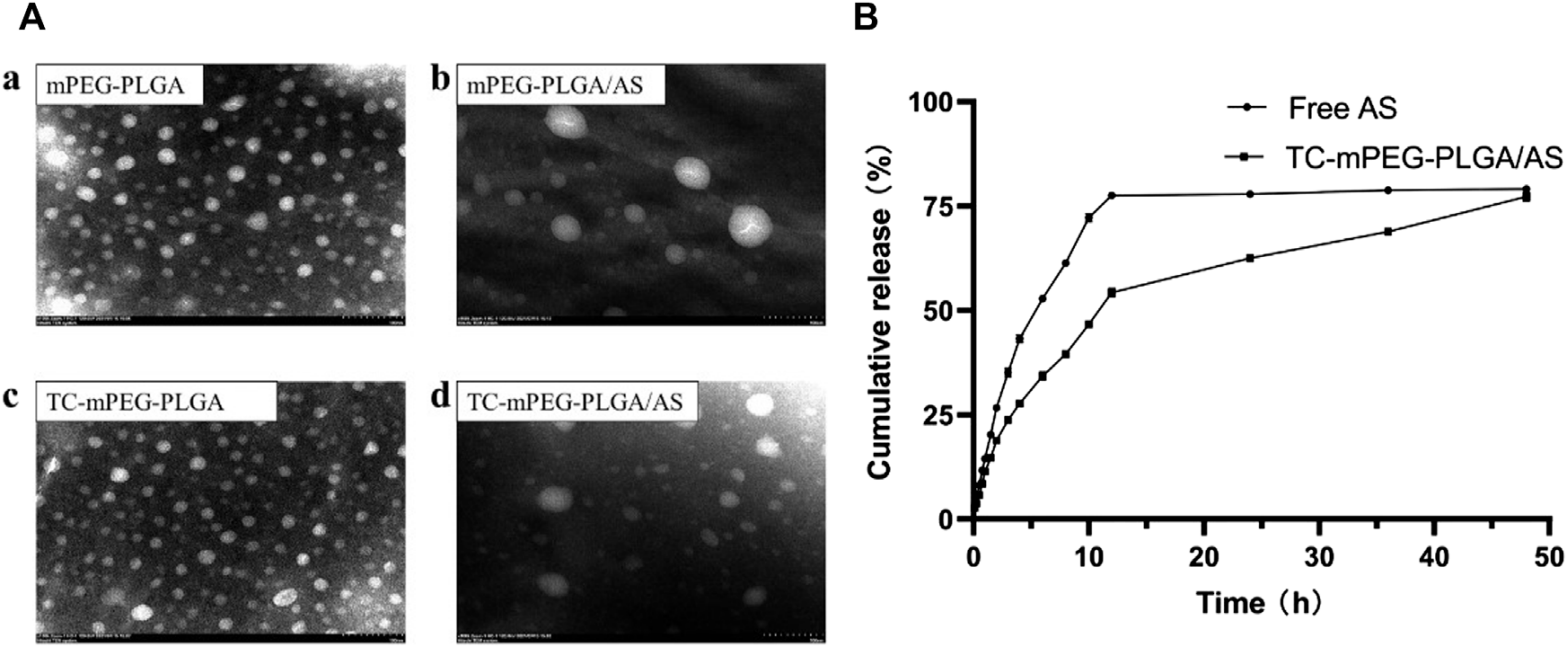
TEM images **(A)** (a, mPEG‒PLGA micelles; b, AS-loaded mPEG‒PLGA micelles; c, TC grafted mPEG‒PLGA micelles; d, AS-loaded TC grafted mPEG‒PLGA micelles) and *in vitro* release behavior in PBS **(B)**. TEM, transmission electron microscopy; AS, astragaloside IV; TC, tetracycline; PBS, phosphate-buffered saline.

Thorough dialysis method has been widely used in polymer micelles prepared for simple operating processors (Nah et al., 1998; Liu et al., 2018; Zhao et al., 2020). We successfully prepared the AS-loaded TC‒mPEG‒PLGA micelle (TC‒mPEG‒PLGA/AS), which was formed spontaneously in aqueous solution. The formed mPEG‒PLGA/AS and TC‒mPEG‒PLGA/AS solutions were opalescent. As shown in Table 1, when the ratio of AS to mPEG‒PLGA or TC‒mPEG‒PLGA was 10:100, the combined result of particle size, zeta potential, and PDI was the most appropriate. The respective mean droplet size, PDI, and zeta potential of mPEG‒PLGA and mPEG‒PLGA/AS were 57.19 ± 0.29 nm, 0.156 ± 0.002 and −10.36 ± 1.13 mV and 64.88 ± 3.59 nm, 0.288 ± 0.027 and −22.59 ± 1.16 mV. Additionally, the mean droplet size, PDI, and zeta potential of TC‒mPEG‒PLGA and TC‒mPEG‒PLGA/AS were 52.63 ± 3.77 nm, 0.268 ± 0.056 and −11.41 ± 0.73 mV and 62.16 ± 2.44 nm, 0.270 ± 0.028 and −19.65 ± 0.74 mV, respectively. No significant change in droplet size was observed after AS was loaded into the micelle. The TEM image in Figure 2A showed that mPEG‒PLGA, mPEG‒PLGA/AS, TC‒mPEG‒PLGA and TC‒mPEG‒PLGA/AS were uniform in size and spherical or elliptical in shape.

**TABLE 1:** The particle size, PDI and zeta potential of different ratio of AS-loaded in mPEG‒PLGA or TC grafted mPEG‒PLGA (*n* = 3).

AS:Ingredient	mPEG-PLGA			TC grafted mPEG-PLGA		
	Particle size	PDI	Zeta potential	Particle size	PDI	Zeta potential
0 mg:80 mg	57.19 ± 0.29	0.156 ± 0.002	−10.36 ± 1.13	52.63 ± 3.77	0.268 ± 0.056	−11.41 ± 0.73
10 mg:80 mg	101.95 ± 2.65	0.207 ± 0.006	−22.54 ± 0.99	109.63 ± 2.06	0.194 ± 0.010	−21.35 ± 0.45
10 mg:100 mg	64.88 ± 3.59	0.288 ± 0.027	−22.59 ± 1.16	52.16 ± 2.44	0.270 ± 0.028	−19.65 ± 0.74
10 mg:120 mg	58.70 ± 1.16	0.265 ± 0.036	−13.06 ± 0.66	51.81 ± 1.71	0.298 ± 0.008	−13.47 ± 1.11
10 mg:140 mg	54.97 ± 1.97	0.336 ± 0.023	−11.48 ± 1.04	49.42 ± 0.80	0.350 ± 0.034	−11.81 ± 0.86

AS, Astragaloside IV; TC, tetracycline; PDI, polymer dispersity index.

### 3.3 Determination of EE and DL

The HPLC analysis of AS *in vitro via* linear regression showed a good linearity within concentration ranging from 0.1 to 100 μg/ml, wherein y = 7,423.9x + 1,541.9 (*R* ^2^ = 0.9971) was the standard curve with x representing AS concentration and y denoting peak area). A relative standard deviation (RSD) of repeatability and precision was observed, while the mean recoveries were over 95%. The EE and DL were determined by separating the free state form of AS from TC‒mPEG‒PLGA/AS. The EE and DL of TC‒mPEG‒PLGA/AS were 93.92 ± 0.34% and 8.72 ± 0.09%, respectively. The affinity between AS and the hydrophobic inner of TC‒mPEG‒PLGA could be an important factor which positively affects drug loading (Liu et al., 2004). Thus, high EE and DL suggest that the hydrophobic alkyl chain of PLGA has a strong affinity for AS.

### 3.4 AS release behavior *in vitro*

*In vitro* release behavior of AS from TC‒mPEG‒PLGA/AS was investigated using the dialysis bag diffusion method. From Figure 2B, the cumulative release of TC‒mPEG‒PLGA/AS was observed to be slower than free AS. Besides, the cumulative release rate of TC‒mPEG‒PLGA/AS (77.25%) was slightly lower than free AS (79.05%) in 48 h. The underlying reason for this phenomenon may be ascribable to potential encapsulation of hydrophobic AS in the micellar core, which might have delayed the release time through diffusion (Gupta et al., 2014). As a result of this, TC‒mPEG‒PLGA/AS exhibited an obvious slow release effect.

### 3.5 Cytotoxicity evaluation

**FIGURE 3:**
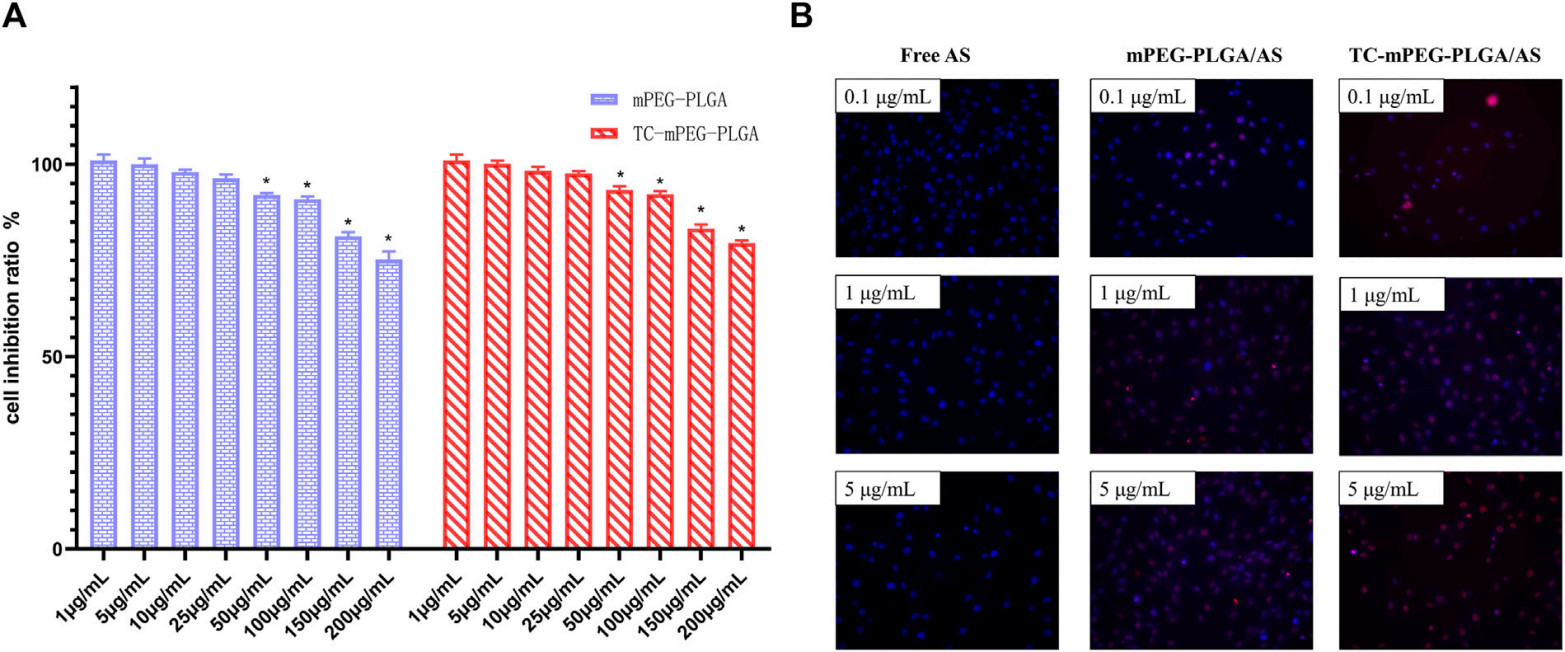
Cytotoxic effects of mPEG‒PLGA micelles and TC grafted mPEG‒PLGA micelles **(A)** and fluorescence images **(B)** of free AS, AS-loaded mPEG‒PLGA micelles, and AS-loaded TC grafted mPEG‒PLGA micelles in femurs. TC, tetracycline; AS, astragaloside IV.

As a prospective drug delivery system, it is expected that TC‒mPEG‒PLGA demonstrates good biocompatibility. Figure 3A shows the findings of an MTT assay that was utilized to investigate the cellular safety of TC‒mPEG‒PLGA/AS. It was found that mPEG‒PLGA and TC‒mPEG‒PLGA were non-cytotoxic to MC3T3-E1 cells with a cell survival rate of around 100% at a concentration range of 1–25 μg/ml. We therefore speculate that mPEG‒PLGA and TC‒mPEG‒PLGA exhibit good biocompatibility in a certain concentration range. Besides, compared with mPEG‒PLGA, TC‒mPEG‒PLGA showed less cytotoxicity at 50–200 μg/ml. Because TC-mPEG-PLGA showed less cytotoxicity at 50–200 μg/ml compared to mPEG-PLGA stably load AS into the micellar core, which consequently might have not influenced the physiological activities of MC3T3-E1 cells (Liu et al., 2012; Song et al., 2016). Thus, TC-mPEG-PLGA can be used to safely deliver AS to bone tissues.

### 3.6 Investigation of uptake of micelles by MC3T3-E1 cells

The uptake of free AS, mPEG‒PLGA, and TC‒mPEG‒PLGA/AS by MC3T3-E1 cells was assessed. In the study, AS internalization into the cells was quantified with ODA–FITC as a label. As displayed in Figure 3B, fluorescence in TC‒mPEG‒PLGA/AS was evidently higher than that of mPEG‒PLGA/AS and free AS. Besides, fluorescence was increased with increased dosage. Based on these results, we postulated that mPEG‒PLGA/AS could improve cellular uptake of AS in MC3T3-E1 cells. The increased AS uptake of mPEG‒PLGA/AS could be attributed to the fact that AS-loaded nanoparticles were more easily absorbed by MC3T3-E1 cells through endocytosis, for the nano size of 52.16 ± 2.44 nm. On the contrary, insoluble free AS was more likely to form larger aggregates, preventing internalization (Song et al., 2018). Normally, endocytosis serves as the major cellular process through which nutrients and other compounds are taken up. Also, this is the process wherein most of nanocarriers are conceived to be taken up. Two categories of endocytosis process are fluids and solutes uptake (pinocytosis) and large particles uptake (phagocytosis). Since this a preliminary study of TC-mPEG-PLGA uptake by cells, we did not ascertain the exact underlying mechanism, notwithstanding. Besides, existing literature suggests lack of data on the uptake mechanisms and intracellular trafficking of polymer micelles remains to be fully elucidated (Nelemans and Gurevich, 2020). Notwithstanding, our future study will comprehensively investigate detail uptake mechanism of TC-mPEG-PLGA.

### 3.7 Ability of micelles to bind to bone mineral

**FIGURE 4:**
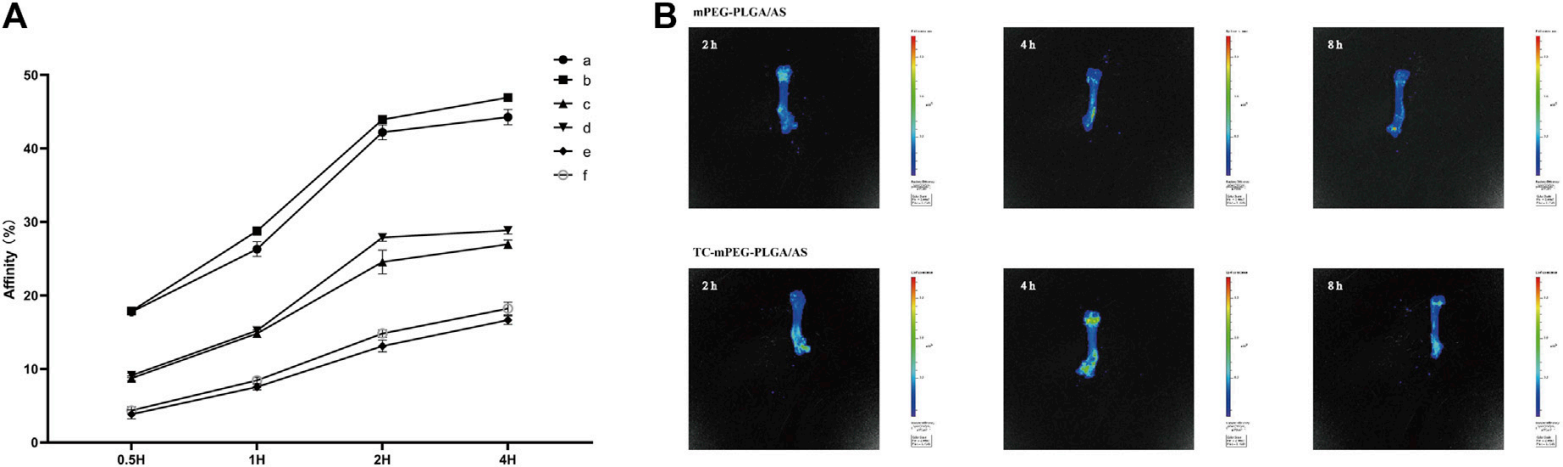
The results of hydroxyapatite targeting ability experiments **(A)** (a, AS-loaded TC‒mPEG‒PLGA micelles containing 5 mg AS; b, AS-loaded TC‒mPEG‒PLGA micelles containing 1 mg AS; c, AS-loaded mPEG‒PLGA micelles containing 5 mg AS; d, AS-loaded mPEG‒PLGA micelles containing 1 mg AS; e, free AS containing 5 mg AS; F, free AS containing 1 mg AS) and a fluorescent *in vivo* imaging of AS-loaded mPEG‒PLGA micelles and AS-loaded TC‒mPEG‒PLGA micelles **(B)**.

Hydroxyapatite is considered the principal constituent of bones in vertebrates. The hydroxyapatite binding test is a general method for testing bone targeting ability *in vitro*. The binding abilities of free AS, mPEG‒PLGA/AS, and TC‒mPEG‒PLGA/AS are shown in Figure 4A. We could clearly observe that the AS readily interacts with hydroxyapatite in the TC‒mPEG‒PLGA/AS group (1 mg: 44.26%, 5 mg: 46.93%), comparable to free AS (1 mg: 26.98%, 5 mg: 28.83%) and mPEG‒PLGA/AS groups (1 mg: 16.65%, 5 mg: 18.22%) within 4 h. This result implies that the grafted TC could significantly improve the bone binding ability of AS and mPEG‒PLGA/AS. The ability of TC to target bone could be attributed to its strong ability to form metal complexes. Thus, TC could replace two PO_4_^3-^ moieties in hydroxyapatite to form a complex with Ca^2+^ (Watkins et al., 2015).

### 3.8 *In vivo* bone target assay

We further explored the bone targeting ability of TC‒mPEG‒PLGA with a fluorescent *in vivo* imaging technique. Fluorescent intensities of mPEG‒PLGA and TC‒mPEG‒PLGA reached the femur within 4 h after they had been injected into the tail vein (Figure 4B). Notably, fluorescent intensities of DiR in the femurs of mice in the TC‒mPEG‒PLGA group were clearly greater than those in the mPEG‒PLGA control group. Therefore, we suggest the bone-targeted capability of TC‒mPEG‒PLGA copolymer through *in vivo* accumulation into the organ, consistent with *in vitro* study.

### 3.9 Estimation of pharmacokinetic parameters in plasma

**FIGURE 5:**
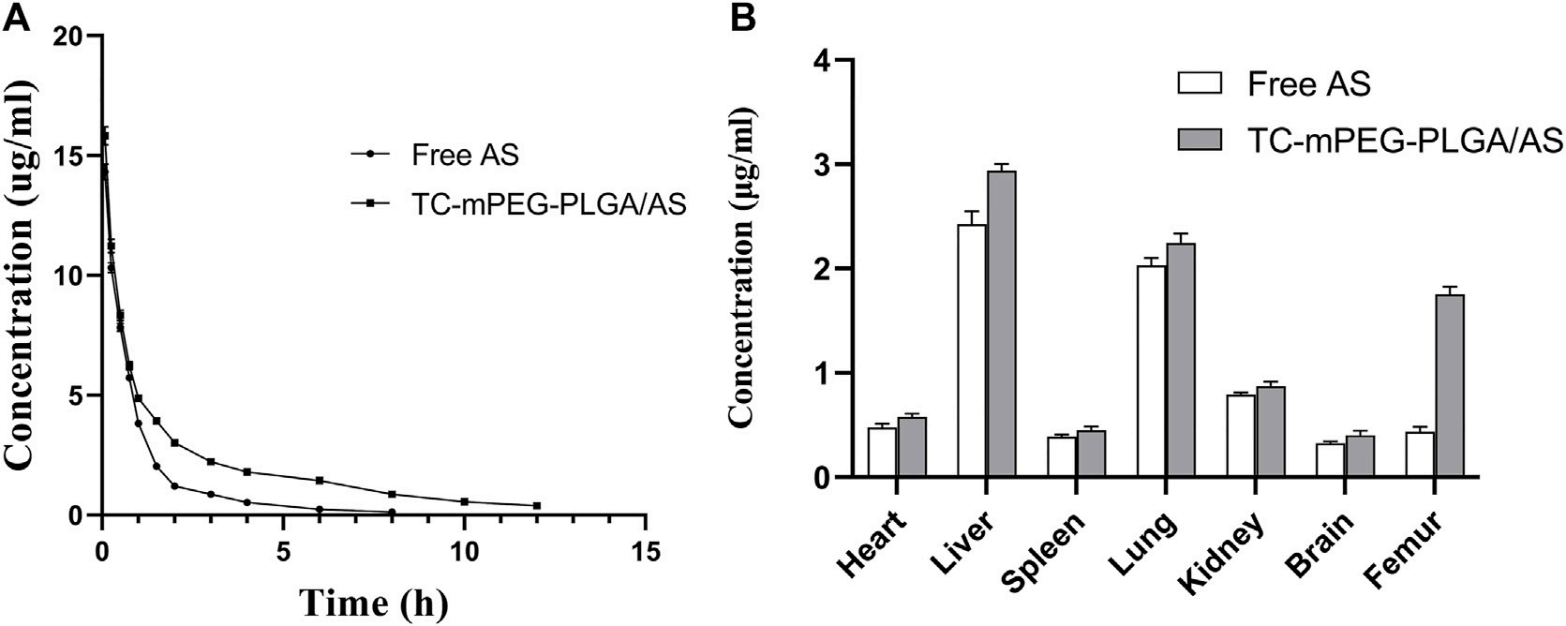
Concentration time profiles in plasma **(A)** and tissue distribution **(B)** of free AS and AS-loaded TC grafted mPEG‒PLGA micelles. TC, tetracycline and AS, astragaloside IV.

The established HPLC standard curve for computing AS content in plasma was linear (y = 0.0126x−0.004, *R* ^2^ = 0.9971), with AS concentration denoted as x, while the ratio of AS peak area to that of baicalein was represented as y). The drug‒time curve of free AS and TC‒mPEG‒PLGA/AS is shown in Figure 5A, wherein it is clearly depicted that the AS concentration in the plasma of rats in TC‒mPEG‒PLGA/AS increased markedly compared to that in free AS, which indicates that the former could promote drug absorption *in vivo*. The pharmacokinetic parameters of free AS and TC‒mPEG‒PLGA/AS, including AUC_t_ (12.912 h μg/ml, 28.260 h μg/ml), MRT (1.369 h, 4.728 h), T_max_ (0.083 h, 0.083 h), C_max_ (14.32 μg/ml, 15.82 μg/ml) and T_1/2_ (0.559 h, 1.094 h) are summarized in Table 2. The relative biological availability (RBA) of TC‒mPEG‒PLGA/AS was 218.9% relative to free AS, which verified that TC‒mPEG‒PLGA could promote AS assimilation into blood. The phenomenon may be attributed to the fact that PLGA polymer nanoparticles had a smaller particle size (52.16 ± 2.44 nm) and a large surface area, which assured their passage through the cell membrane (Beletsi et al., 2005; Yallapu et al., 2014). Belesti et al. found that nanoparticles composed of PLGA and PEG‒PLGA polymers had long blood circulation, which is consistent with our research (Wong et al., 2007).

**TABLE 2:** Pharmacokinetic parameters of free AS and AS-loaded TC grafted mPEG‒PLGA after tail vein (n = 6).

Parameters	Free AS	TC-mPEG-PLGA/AS
C_max_ (μg/ml)	14.32	15.82
T_max_ (h)	0.083	0.083
t_1/2_ (h)	0.559	1.094
MRT (h)	1.369	4.728
AUC_t_ (h μg/ml)	12.912	28.260

AUC_t_, area under the concentration time curve; C_max_, maximum concentration of drug; T_max_, time to attain C_max_; t_1/2_, time needed for initial AS, concentration to reduce to half; MRT, mean residence time; AS, Astragaloside IV; TC-mPEG-PLGA/AS, and AS, loaded tetracycline grafted mPEG-PLGA, micelle.

### 3.10 Tissue distribution

According to tissue distribution results, the TC‒mPEG‒PLGA could significantly alter AS accumulation compared with free AS. As shown in Figure 5B, compared with free AS, TC‒mPEG‒PLGA demonstrated an obvious accumulation behavior in the femur. The notable femur targeting ability could be ascribed to the capability of TC to form metal complexes with bone ingredients. Thus, the excellent femur targeting property of TC‒mPEG‒PLGA may improve the reported osteoporotic activity of AS. Besides, the TC‒mPEG‒PLGA/AS was found to exhibit a liver accumulation effect. This phenomenon may be attributed to the uptake and phagocytic mechanism of macrophage cells in the liver (Pade and Stavchansky, 1998). After 4 h, the AS concentration in all organs of mice in the TC‒mPEG‒PLGA group was still higher than in the free AS group, which demonstrated an obvious prolonged absorption effect. The amount of AS distributed in the blood and each organ is all lower than that in TC-mPGE-PLGA/AS group. Because AS is insoluble in water and has poor oral bioavailability, while TC-MPGE-PLGA/AS increased the water solubility of AS which could promote the absorption of AS in the body (Lin et al., 2014). In this work, TC-mPEG-PLGA could differentially promote AS absorption *in vivo* comparable to free AS because of the possibility of TC-mPEG-PLGA diffusing and permeating the intestinal mucosa (Wang et al., 2013). Since the PEG molecules that coat the surfaces of TC-mPEG-PLGA is hydrophilic, it is possible it could promote the dissolution of the micellar system into cells of the epithelium, which subsequently increased the permeability and absorption of the micelle. Besides, the smaller particle size and negative zeta potential might have played a role in the transport of TC-mPEG-PLGA across intestinal mucosa as stated elsewhere (Wang et al., 2013)).

### 3.11 *In vivo* pharmacodynamics study

**FIGURE 6:**
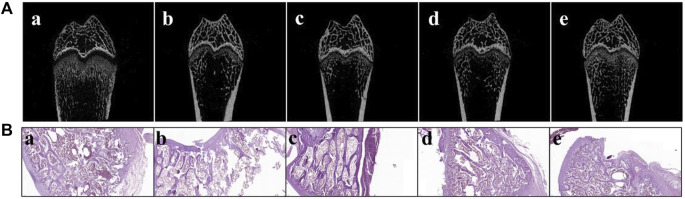
Micro-computed tomography scan **(A)** and histopathological photomicrographs **(B)** of femurs from ovariectomized (OVX) rats. (a. SHAM, b. OVX, c. OVX + ES, d. OVX + AS group, and e. OVX + AS-loaded TC‒mPEG‒PLGA micelles groups). The ES, AS, and AS-loaded TC‒mPEG‒PLGA micelles were administered at a dose of 0.5 mg/kg/2 days via the tail vein. OVX, ovariectomized; AS, astragaloside IV; ES, estradiol; TC, tetracycline.

The femur BMD in the SHAM group (0.121 ± 0.006) was distinctly different from that in the OVX group (0.057 ± 0.001) (*p* < 0.001, Table 3), which demonstrates that the osteoporotic model was successfully established in ovariectomized rats. After treatment for 8 weeks, the femurs of all of the animals were collected. Rats in free AS showed a certain mitigative effect on osteoporotic in ovariectomized rats comparable to the OVX group (*p* < 0.05). Moreover, compared to the OVX group, femur BMD in rats of the OVX + AS, OVX + ES, and OVX + TC‒mPEG‒PLGA/AS groups increased by 12.9, 62.5, and 62.7%, respectively. Besides, a structure model index (SMI) was also detected, which reflects the characteristics of bone trabecular plate-like and rod-like structures. When osteoporosis occurs, trabecular bone changes from a plate-like structure to a rod-like structure while SMI values increase. Besides, the tendency of SMI changes was consistent with that of BMD, which further confirmed the therapeutic effectiveness of TC‒mPEG‒PLGA/AS on osteoporosis. We further analyzed the bone micromorphological indicators of the femur, including BV/TV, Tb.N, Tb. Sp, and Tb.Th, wherein the results are presented in Table 3, amid confirmation of our conjecture again. As shown in Figure 6A, 3D reconstruction of the femur showed the trend more clearly and intuitively. The results hinted that TC‒mPEG‒PLGA could target bone and promote accumulation of AS in bone tissue. Further, TC‒mPEG‒PLGA could improve the curative effects of AS on osteoporosis. As a result, TC‒mPEG‒PLGA could be used as a novel delivery system for molecules with anti-osteoporotic activity, potentially lowering therapeutic doses.

**TABLE 3:** Morphological parameters of the femur of rats in each group after being ovariectomized (*n* = 3).

Group	BV/TV (%)	Tb.Th (mm)	Tb.N	Tb.Sp	SMI	BMD
SHAM	16.254 ± 0.467	0.076 ± 0.004	2.234 ± 0.096	0.462 ± 0.003	1.823 ± 0.029	0.121 ± 0.006
OVX	8.654 ± 0.341	0.067 ± 0.003	1.183 ± 0.010	0.71 ± 0.013	2.228 ± 0.091	0.057 ± 0.001
OVX + ES	12.91 ± 0.703	0.074 ± 0.002	1.943 ± 0.063	0.48 ± 0.016	1.793 ± 0.052	0.093 ± 0.003
OVX + AS	9.951 ± 0.100	0.076 ± 0.002	1.441 ± 0.026	0.60 ± 0.018	1.847 ± 0.032	0.064 ± 0.003
OVX + TC-mPEG-PLGA/AS	12.171 ± 0.454	0.078 ± 0.001	2.042 ± 0.068	0.562 ± 0.024	1.812 ± 0.016	0.093 ± 0.002

OVX, ovariectomized; AS, Astragaloside IV; ES, estradiol; TC, tetracycline; SMI, structure model index; BMD, bone mineral density.

### 3.12 Histopathological analysis

H&E staining was carried out after decalcification on the lower segment of the right femur. As indicated in Figure 6B, comparable to the SHAM group, the characteristics of the lower segment of the femur in the OVX group were as follows: the epiphysis line was significantly thinner with a loose trabecular arrangement, while the number of trabecular bones was significantly reduced. The arrangement was not regular with the occurrence of local fractures, which indicates marked osteoporosis, coupled with abundant fatty yellow bone marrow replacement by red bone marrow. Compared with the model group, the epiphysis line was thickened in the TC‒mPEG‒PLGA/AS group, while the lower trabecular bone was closely arranged, with a significant increased number and an obvious regular arrangement, thereby suggesting that the status of osteoporosis was substantially improved. These results are consistent with the BMD value that was estimated earlier in this work.

## 4 Conclusion

In this study, TC‒mPEG‒PLGA was successfully developed, while its bone targeting ability and anti-osteoporotic effects were confirmed. Optimized TC‒mPEG‒PLGA/AS had smaller sized droplets, which exhibited a slow-release effect *in vitro*. In cell evaluation tests, TC‒mPEG‒PLGA demonstrated greater ability to uptake by MC3T3-E1 cells and biocompatibility with fewer cytotoxic effects. Besides, due to excellent affinity between TC and hydroxyapatite, TC‒mPEG‒PLGA could enhance the bone targeting ability of AS in hydroxyapatite binding tests. *In vivo* bone target assays revealed that TC‒mPEG‒PLGA was more likely to accumulate in bone than mPEG‒PLGA. In terms of pharmacodynamics, TC‒mPEG‒PLGA potentially enhanced the anti-osteoporotic effect of AS. Therefore, TC‒mPEG‒PLGA may act as an effective delivery system for molecules with the potential to treat osteoporosis.

## Data Availability

The raw data supporting the conclusion of this article will be made available by the authors, without undue reservation.
